# Diverse Temperate Coliphages of the Urinary Tract

**DOI:** 10.3390/v18020179

**Published:** 2026-01-29

**Authors:** Haley Atkins, Natalie Stegman, Catherine Putonti

**Affiliations:** 1 Bioinformatics Program, Loyola University Chicago, Chicago, IL 60660, USA; 2 Biology Department, Loyola University Chicago, Chicago, IL 60660, USA

**Keywords:** coliphages, urinary tract, *E. coli*

## Abstract

While *Escherichia coli* can be found in the bladders of females without lower urinary tract symptoms, its presence is often associated with urinary tract infections (UTIs). The genomic plasticity of *E.* coli, including urogenital strains, is largely shaped by the integration of prophages. Although genomic and metagenomic analyses of urinary *E. coli* and the urinary microbiome suggest that prophages are abundant, many represent uncharacterized species. Sequence analysis suggests that these prophages represent temperate phages. This study aimed to fill this gap, isolating and characterizing temperate phages from urinary *E. coli* strains. We assessed phage host range across a panel of urinary isolates, providing a critical first step for future work investigating their putative role in shaping *E. coli* populations within the urinary community. In total, 20 temperate urinary phages were evaluated. Phage morphology and genic content of these phages were determined via transmission electron microscopy (TEM) and whole-genome sequencing, respectively. Together, these analyses provide insight into the diversity, infectivity, and genomic composition of temperate coliphages from the female urinary tract.

## 1. Introduction

*Escherichia coli*, a common inhabitant of the human gut, is the most frequently identified bacterial species associated with urinary tract infections (UTIs) in females [1]. *E. coli* is also frequently detected in the bladders of females without urinary symptoms [2,3,4]. The species’ genetic diversity, shaped by their environment and mobile genetic elements, contributes to their ability to adapt to and persist in both the nutrient-rich environment of the gut, as well as the nutrient-limited environment of the bladder [5,6]. One of the most significant contributors to *E. coli* genome plasticity is the presence of prophages [7,8,9,10]. Prophages are abundant in *E. coli*, with most *E. coli* genomes encoding for more than one prophage sequence [5,6,9,10,11,12,13]. The same is true for *E. coli* strains isolated from the urinary tract; recent genomic studies consistently found that the majority of urinary *E. coli* strains also include one or more prophage sequences [14,15].

While cryptic prophages, i.e., phages that have integrated and are unable to be induced into the lytic cycle, do not actively produce new virions, they often act as hotspots of recombination within *E. coli*, contributing to genome plasticity [16]. Temperate phages, or prophages capable of entering the lytic life cycle, often benefit their host. They can accelerate bacterial evolution via horizontal gene transfer (HGT), provide immunity against other phages (superinfection immunity) [17], and confer phenotypic benefits (lysogenic conversion) (see reviews [18,19]). *E. coli* temperate phages are among the most thoroughly characterized phages, with phage Lambda serving as a model system for lysogeny and phage-host dynamics since the 1950s [20]. Lambdoids are among the most frequent prophages of *E. coli* [21]. A recent study found an association between lambda phage orientation and (i) genome location of integration and (ii) spontaneous lysis [22]. Despite the benefits of lysogeny, it comes with a cost—excision (induction) of the prophage can cause bacterial host cell death. While detrimental for the individual bacterium, it can be beneficial for the population [23].

Within the human GI microbiota, where temperate phages predominate [24,25,26,27], associations between temperate phages and GI health have emerged (see review [28]). Microbial interactions and external factors can drive prophage shifts from lysogenic to lytic life cycles in the gut, thus modulating the microbial community [29]. In comparison to the gut, the urinary microbiome is not well characterized. Nevertheless, virome studies have found that phages are more abundant than eukaryotic viruses (see review [30]) and lysogeny is widespread (86%) [14]. Our prior analyses of urinary bacterial genomes [14] and urinary *E. coli* genomes [15] suggest that these prophage sequences are temperate phages. This is further supported by studies that have isolated temperate phages from urinary bacteria [15,30,31,32]. However, characterization of the diversity of temperate phages within the urinary tract has yet to be conducted. A recent study suggests that temperate phages may be contributing to uropathogen colonization [33]. Characterization of these phages is a critical first step in understanding the role of temperate phages within this community and their association (if any) with urinary tract symptoms.

Given the high prevalence of prophages in *E. coli* genomes, this study aimed to isolate and characterize temperate phages from urinary *E. coli* isolates. Functional temperate phages were examined to assess their host range, morphology, and genetic composition. Host range assays were performed to evaluate which urinary *E. coli* strains a given phage can infect. Transmission electron microscopy (TEM) was used to visualize phage morphology based on structural characteristics. In parallel, whole-genome sequencing of the phages provided detailed information about their genetic architecture, lysis/lysogeny genes, and taxonomy. Together, these analyses offer a view of temperate coliphages in the urinary microbiota.

## 2. Materials and Methods

### 2.1. Prophage and CRISPR Prediction

The 66 strains of *E. coli* previously sequenced by our lab [3] were evaluated for the presence of prophages using the tool PHASTER [34]. CRISPR/Cas detection was performed using CRISPRCasFinder with the parameters “general” and “unordered,” which allow a permissive search for CAS genes within contigs respectively [35]. The strains and the number of predicted prophage sequences and CRISPR spacers are listed in Supplemental Table S1.

### 2.2. Phage Induction

The 66 strains were used for phage induction (Supplemental Table S1). From our freezer stocks, these bacteria were streaked on LB 1.7% agar plates and incubated overnight at 37 °C. Next, 3 mL of LB was inoculated with a single colony from a plate and grown overnight, with shaking at 37 °C. The laboratory strain *E. coli* C was grown under these same conditions. Each culture of a urinary *E. coli* strain was filtered with a 0.22 μm syringe filter. The filtrate was spotted (10 μL) onto lawns of *E. coli* C. Each lawn consisted of 500 μL of overnight *E. coli* C culture + 3 mL of soft LB agar (0.7% agar) mixed and spread onto an LB agar plate. The spot plates were incubated overnight at 37 °C. Clear plaques were harvested into 0.7% saline, vortexed, and filtered. The filtrate was again plated, this time as pour plates (100 μL filtrate + 3 mL soft LB agar), whereupon individual plaques were harvested. This process of plaque purification was repeated at least three times.

### 2.3. Phage DNA Extraction, Sequencing, Assembly, and Annotation

Phage DNA from harvested plaques was extracted with the Zymo Quick DNA Viral Kit. Two steps were modified in the manufacturer’s protocol: (1) 300 μL of sample and 1200 μL of Viral DNA Buffer were used and (2) 30 μL of elution buffer was used. The concentration of the DNA was measured via Qubit fluorometer. DNA was sent to SeqCoast Genomics, LLC (Portsmouth, NH, USA) for library preparation and sequencing. Samples were prepared for whole-genome sequencing using an Illumina DNA Prep tagmentation kit and unique dual indexes. Sequencing was performed on the Illumina NextSeq2000 platform (San Diego, CA, USA) using a 300-cycle flow cell kit, producing 2 × 150 bp paired-end reads. Genome assembly and annotation were performed using the Bacterial and Viral Bioinformatics Resource Center (BV-BVRC) genome assembly and annotation service (v.3.49.1) [36]. Genome assembly was conducted via BV-BRC using the SPAdes assembly strategy (SPAdes v4.0) [37]. Annotation was performed using the Genome Annotation tool in BV-BRC with the “Bacteriophages” annotation recipe.

### 2.4. Phage Host Range

To assess the host range of the isolated phages, spot tests were performed on the *E. coli* C laboratory strain and the previously described 66 *E. coli* strains of Garretto et al. [3] using the same methods as described previously. Following incubation, plates were examined for zones of lysis. Furthermore, dilutions were plated to assess phage efficacy. Clear zones were recorded as strong lytic activity (plaque formation), zones with no evidence of lytic activity (no plaque), or incomplete zones (“cloudy” plaques) as partial lysis or incomplete lysogeny. Supplemental Figure S1 shows an example of one of these dilutions in which the plaques are not clear but rather appear cloudy.

### 2.5. Transmission Electron Microscopy (TEM)

For TEM analysis, phages were prepared by applying filtered cell-free supernatant to thin carbon-coated copper grids and allowing them to adsorb onto thin carbon films for 2 min. Then, the films were negatively stained with 2% aqueous uranyl acetate for 1 min. Samples were observed using a JEOL 1400 Flash TEM (Boston, MA, USA).

### 2.6. Phage Genome Analysis

Phage genome sequences were first queried against the nr/nt database, restricting the search to viruses (taxonomy ID: 10239). Sequences also were examined using ICTV’s TaxaBLAST (version VMR_MSL40.v1.20250307.3437cb9) [38]; the closest species exemplar sequence was then retrieved from NCBI and compared to the phage genome sequence via VIRIDIC, using the web version of the tool with default parameters [39]. Peduovirus genomes were compared with clinker [40], using the web version of the tool. Default parameters were used with the following exceptions: “Show only best links” was selected and the identity threshold was set to 0.7. Integrase amino acid sequences were extracted from annotations and aligned using MAFFT v7.490 [41] using default parameters via Geneious Prime v2024.0.7. A phylogenetic tree was derived using FastTree v2.1.11 [42], again using default parameters via Geneious Prime, and visualized using iTOL v7 [43]. Lastly, genomes were examined for Anti-CRISPR (Acr) genes using AcrHub [44]. Phage genomes were individually uploaded to the server, and all three (PaCRISPR, AcRanker, and HMM-based predictor) were selected, specifying the “For normal use” option.

## 3. Results

Sixty-six urinary *E. coli* strains were grown and tested for spontaneous induction; these strains have been previously characterized by our lab [3] and each genome was examined for the presence of prophages (Supplemental Table S1 and Supplemental File S1). Filtered cell-free supernatants were plated on the laboratory strain *E. coli* C, as described in the Methods, and plaques were purified. In total, 20 temperate phages were successfully induced. While the bacterial strains tested included >1 predicted prophage (Table 1), through plaque purification, a single phage was isolated from each lysogen. (The predicted prophage sequences from the draft genomes for the strains in which a phage was isolated are provided in Supplemental File S1.) These temperate phages were from *E. coli* strains isolated from females with and without lower urinary tract symptoms and include phylogenetically distinct *E. coli* strains, representative of phylogroups A, B1, B2, D, and F.

### 3.1. Host Range Analysis

**Figure 1:**
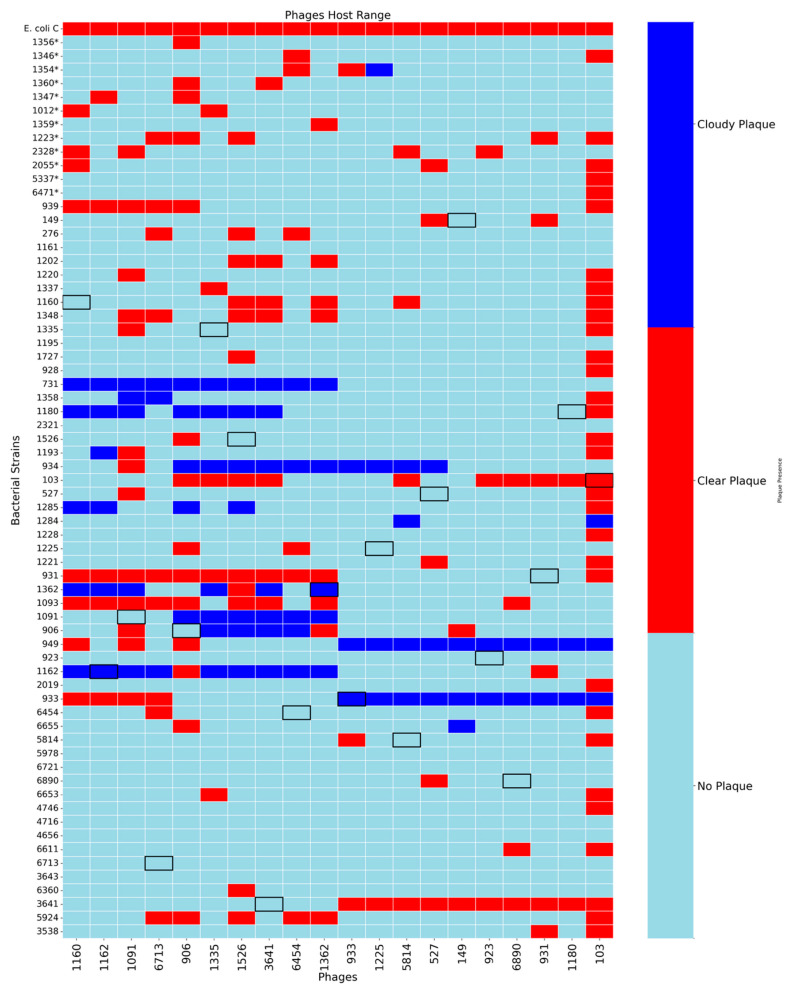
Phage host range across clinical urinary *E. coli* isolates and lab strain *E. coli* C. The heatmap shows the outcome of spot assays for each phage-bacterium pair. Red squares indicate clear plaques, representing productive lytic infection. Dark blue indicates cloudy plaques, suggestive of incomplete lysogeny. Light blue indicates no visible plaque, suggesting no infection or complete resistance. Bacterial strains with no prophages are denoted by an asterisk (*) and listed in the top rows. Black boxes throughout the heatmap represent a phage infecting the host from which it was isolated. (Beyond ordering by *, there is no specific ordering of either bacteria or phage in the figure).

The host range of each phage was assessed using spot assays on the full panel of 66 urinary *E. coli* strains and the well characterized laboratory strain *E. coli* C. Plaque phenotypes were visually classified as either clear or cloudy to indicate complete versus partial lysis, respectively (Figure 1). (An example of a “cloudy” plaque can be seen in Supplemental Figure S1.)

Across all 20 phages tested, we observed considerable variability in host range, with no single phage capable of infecting all 66 urinary bacterial strains. The number of susceptible hosts per phage ranged from as few as 2 to as many as 32. Phage 103 showed the broadest host range, lysing 32 out of the 66 tested urinary strains (48.5%), indicating a capacity for cross-strain infectivity. Other phages with moderately broad host ranges include phage 906 (14/66, 21.2%), phage 109 (13/66, 19.7%), and phage 3461 (13/66, 19.7%). Of the 20 phages examined, 6 phages (30%) were capable of lysing 10 or more strains. Most phages, however, exhibited more narrow host ranges. Phages 1160 and 3461 each lysed 7 strains, while phages 1162 and 527 lysed 5 strains. Several phages showed extremely limited infectivity, including phages 1225, 5814, and 6890 each of which lysed only 2 strains, and phages 149 and 1180, which infected only 3 strains.

Superinfection immunity is common among *E. coli* lysogens [45,46,47,48,49,50,51] and could explain the limited lysis observed; 54 of the 66 strains tested contain prophages themselves. Only 12 of the urinary *E. coli* strains are not predicted to encode for a prophage (UMB1356, UMB1346, UMB1354, UMB1360, UMB1347, UMB1012, UMB1359, UMB1223, UMB2328, UMB2055, UMB5337, and UMB6471) (Figure 1, indicated with an asterisk). These 12 strains represent the only unbiased substrate for detecting lysis uninfluenced by prophage-mediated defense. For example, phage 103, which lysed 33 *E. coli* strains overall, was able to lyse 5 of the 12 prophage-free strains (UMB1346, UMB1223, UMB2055, UMB5337, and UMB6471). Phages 1160, 1162, and 931 showed more instances of lysis on prophage-free strains rather than lysogens, suggesting that they may be particularly susceptible to superinfection exclusions. Phages with very limited host range (phages 1225, 5814, and 6890) did not infect any of the prophage-free strains, indicating inherently narrow tropism, integration into these bacterial hosts, or complete inhibition by intracellular defenses. Notably, when we examine phage infectivity across these prophage-free strains, some phages that otherwise showed limited host range produced plaques more consistently.

### 3.2. TEM Imaging

**Figure 2:**
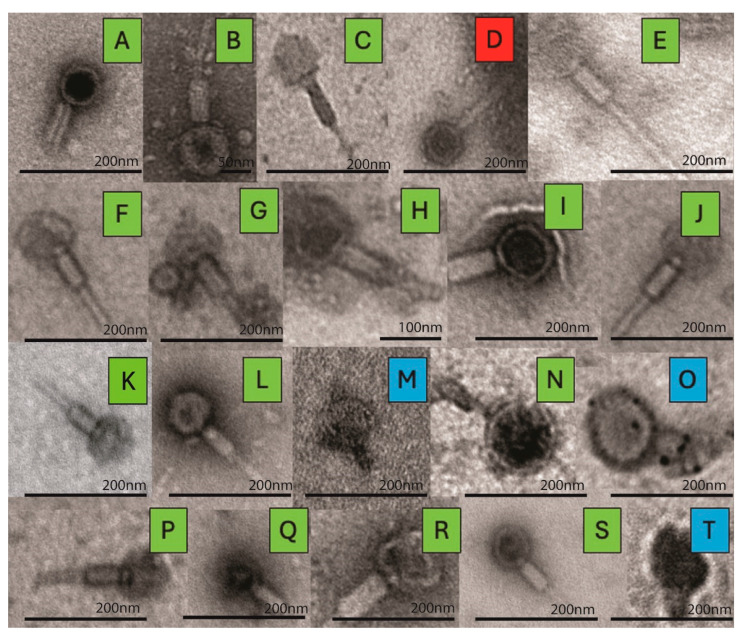
TEM of phages isolated from urinary *E. coli* lysogens. Representative TEM images of 20 phages showing the structural diversity across the Caudoviricetes class. Each color corresponds to a different morphology: green for myoviruses; blue for podoviruses; red for siphoviruses. Each panel (**A**–**T**) depicts a distinct phage isolate: (**A**) 6890, (**B**) 5814, (**C**) 1526, (**D**) 1180, (**E**) 1335, (**F**) 1162, (**G**) 906, (**H**) 1160, (**I**) 933, (**J**) 923, (**K**) 149, (**L**) 1091, (**M**) 103, (**N**) 931, (**O**) 527, (**P**) 6713, (**Q**) 3461, (**R**) 6454, (**S**) 1225, and (**T**) 1362.

Phage morphology was characterized using TEM, which revealed distinct, structural features characteristic of the Caudoviricetes class (previously known as Caudovirales) of tailed phages with double-stranded DNA genomes [52]. In Figure 2, TEM images of the 20 isolated induced phages are shown. Here we can see that most of the isolated phages—6890, 5814, 1335, 1162, 906, 1160, 933, 923, 149, 109, 931, 6712, 3461, 6454, and 1225—exhibited features characteristic of myoviruses, with notably rigid, contractile tails. This group includes both broad host range phages (906, 1162, and 1335) and narrow host range phages (933 and 1225), highlighting the variety of host ranges within this morphology.

A single siphovirus phage was detected, phage 1526, which exhibited a long, flexible, non-contractile tail. This family is often associated with temperate phages, and while some siphoviruses can infect diverse hosts, they typically display narrower host ranges [53,54]. However, phage 1526 exhibited a moderate host range, infecting 13 of 55 tested strains (19.7%). Three phages—103, 527, and 1362—were identified as likely podoviruses, possessing short, non-contractile tails. Interestingly, phage 103 displayed the broadest host range in this study, lysing 32 of 66 strains (48.5%), whereas 527 and 1362 exhibited much narrower ranges.

### 3.3. Genome Sequence Analysis

Next, the induced phages were sequenced, and their genomes assembled and annotated. Details about their genome sequences can be found in Supplemental Table S2. Each phage genome was then queried against all characterized virus sequences in GenBank via BLAST (Table 2). In cases in which the genome sequence identified via this BLAST search had an assigned tail morphology, we have included this in Table 2. While no morphological information was available for 12 of these GenBank records, we should note that two of the phages identified via TEM as podoviruses (phages 527 and 1362) exhibit the greatest sequence similarity to known podoviruses; phage 103, also identified as a podovirus, exhibits the greatest sequence similarity to an uncharacterized phage. While TEM imaging suggests that phage 906 is a myovirus (Figure 2), its sequence is most similar to a characterized podovirus (query coverage = 56%, percent identity = 96.05%).

As Table 2 shows, only nine of the phage genomes were most similar to characterized phages. Next, we compared the sequences to the International Committee on Taxonomy of Viruses (ICTV) species exemplar sequences. (Due to the fragmentation of the sequences for phages 1335 and 6713, we have excluded them from this analysis.) The intergenomic similarity reported in Table 2 and illustrated in Supplemental Figure S2 indicates that all of the phages isolated are representatives of new species, as none exceed the 95% threshold set forth by the ICTV [52]. However, we can confidently assign six of the induced phages to a genus, as the intergenomic similarity exceeds the 70% threshold [52]. Coincidentally, they are all members of the genus *Peduovirus*. None of the other phages meet this genus threshold, although phages 933 and 6890 are close. This suggests representatives of putative new genera.

**Figure 3:**
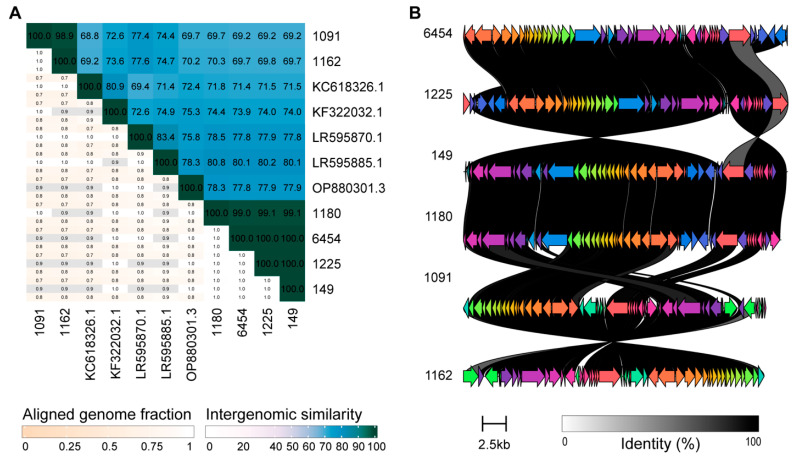
Comparison of phage isolate genome sequences similar to peduoviruses. (**A**) The upper triangle lists the intergenomic similarity (%) while the lower triangle lists the fraction (between 0 and 1) of the genome that aligned. Three measures are provided for the genome alignment: the aligned fraction genome 1 (row genome), genome length ratio (for the two genomes in the pair), and aligned fraction genome 2 (column genome). (**B**) Annotated coding regions are indicated by arrows, pointing in the direction of their orientation. Homologous genes are displayed in the same color and connected between the four phage sequences via lines. These lines are shaded from light to dark based on the amino acid sequence identity. Arrows shaded gray do not have homologs in any of the other genomes.

Further investigation of the phages identified as members of the genus *Peduovirus* was conducted. As shown in Figure 3A, phages 1180, 6454, 1225, and 149 are representatives of the same species; their intergenomic similarity exceeds 99%. Likewise, phages 1091 and 1162 represent the same species (intergenomic similarity = 98.9%). The inclusion of five ICTV species exemplar sequences in Figure 3A confirms their membership in the genus. We can thus conclude that the induced prophages represent two new *Peduovirus* species. Significant amino acid similarity is observed between the coding regions of all six phages (Figure 3B).

**Figure 4:**
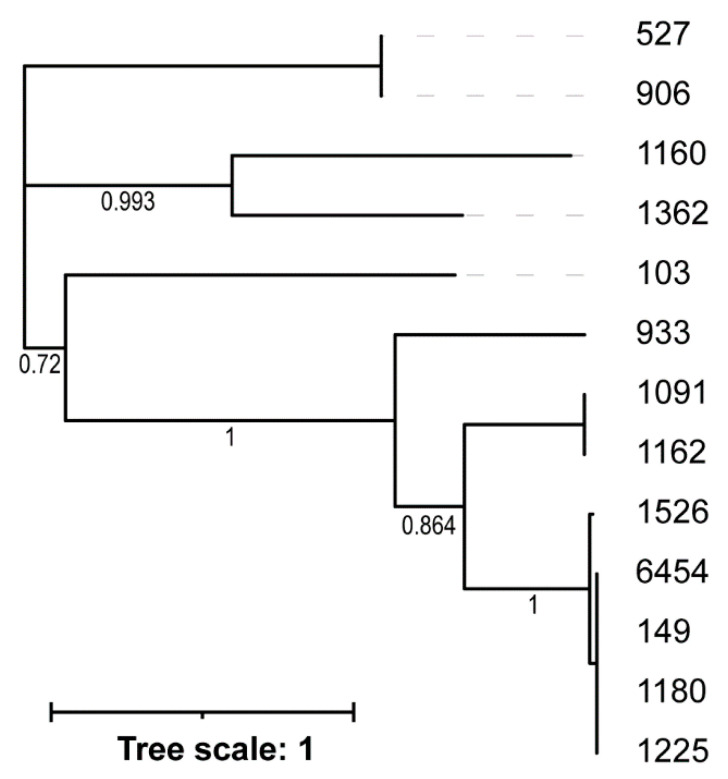
Tree of identified integrase amino acid sequences. Branch supports are shown.

The annotated phage sequences were further examined, identifying integrase coding regions in 13 of the 20 genome sequences. No integrase was identified for phages 923, 931, 3641, 5814, 6890, nor the two fragmented assemblies. All 13 integrases are predicted to be tyrosine recombinases. A phylogenetic tree of these integrase amino acid sequences was derived (Figure 4). Of note is the placement of phage 1526, which was similar to—although not meeting the genus threshold—*Peduoviruses* (Table 2). Phages 527 and 906 form a distinct clade, which parallels their distinction both visually as podoviruses and their similarity to sequences of the genus *Lederbergvirus*.

Given the presence of CRISPR spacers within the urinary *E. coli* strains tested for permissivity to the induced phages (Supplemental Table S1), the phage genomes were examined for Anti-CRISPR (Acr) proteins. As shown in Supplemental Table S3, all but phage 933 contained at least one predicted Arc. Note, no very close homologs to experimentally verified Acrs were identified. Further investigation into the predicted genes is needed to ascertain if these coding regions assist with overcoming the bacterial host defense system.

## 4. Discussion

Previous work by our group has isolated temperate phages from urinary isolates, e.g., [15,30,31,57]. With the recent evidence suggesting that temperate phages may contribute to uropathogen colonization [33], we focused our efforts on further understanding coliphages. *E. coli* colonizing the urinary tract can itself be diverse, representing most of the phylogroups for the species, both in healthy individuals and those with UTIs [3,58,59,60,61]. Given this diversity, we previously investigated urinary *E. coli* genomes, finding prophages predicted to represent all three phage tail morphologies [15]. Here, we were able to induce and reliably replicate temperate phages from diverse urinary *E. coli* strains (Table 1).

Our host range analysis of the 20 induced prophages shows significant variation. Most of the induced phages could not infect the host from which they were induced; only phages 1162 and 933 produced cloudy plaques and phage 103 was able to completely lyse its source strain (Figure 1). Notably, six phages (1225, 5814, 149, 6890, 1161, and 1012) were restricted to being able to successfully lyse just 2–4 hosts. However, the absence of observable plaques should not be interpreted as the inability to infect the bacterium. In many cases, temperate phages may adsorb and inject their DNA successfully into the bacterial cell but enter the lysogenic cycle instead of initiating lysis, resulting in no visible plaque formation [62]. Alternatively, even when the lytic cycle is attempted, bacterial defenses can mask the true breadth of some phages’ ability to infect a host cell [63]. These defenses, including restriction modification (RM) systems, CRISPR-Cas immunity, or prophage-mediated superinfection exclusion, can interfere with phage replication and abort the infection [64,65,66]. While prior studies have found the CRISPR-Cas and other “anti-phage” defenses abundant among *E. coli* [67], 30 of the 66 *E. coli* strains tested here did not encode for the CRISPR-Cas system (Supplemental Table S1). Phage defenses against the CRISPR-Cas system, also known as anti-CRISPR genes, have been identified for several species, although a full catalog of these genes across the prokaryotic tree of life has yet to be generated [68]. Anti-CRISPR genes were predicted in all but one of the induced phage genomes, although no genes homologous to experimentally verified genes were identified (Supplemental Table S3). Further investigation of these predicted Acr genes and other defense systems that may be encoded within these phage genomes is needed.

The six phages (1091, 1162, 149, 1180, 6454, and 1225), which most closely resembled the well-studied family of P2-like temperate myoviruses [69], varied in their host-ranges (Figure 1). Phage 1225 only fully lysed two of the *E. coli* strains tested and phages 149 and 1180 fully lysed three. Phage 1162 fully lysed six and phage 6454 fully lysed seven *E. coli* strains. This is in contrast to phage 1091, which fully lysed 14 of the *E. coli* strains tested. Even phages representative of the same species varied in the number of *E. coli* strains lysed, with phage 1091 exhibiting a much broader host range than 1162. The bacterial strains lysed also varied. The variation in host range observed fits with prior studies. Host range can vary between closely related phages in the same species [70,71,72]; in fact, a single “well-placed” mutation can result in dramatically different host ranges for two phages [73]. It should be noted that phage 1180, which was initially predicted to be a siphovirus (Figure 2), genome sequencing suggests that the induced phage is a *Peduovirus*. Because induction was repeated for this particular *E. coli* strain, it could be that we have imaged one temperate phage—a siphovirus—while sequencing another—a *Peduovirus*.

In a recent study of coliphages from fecal samples of 1 year old children, lytic phages had a broader host range than temperate phages when tested against *E. coli* isolates from these same samples; temperate phages were able to lyse at most 9% of the 75 *E. coli* isolates tested [74]. In contrast, we identified temperate coliphages with substantially greater host range, most notably phage 103, which successfully lysed nearly half of the 66 urinary *E. coli* strains. Phage 103 sequencing and comparison to characterized phages indicate that this phage has yet to be isolated, showing the greatest sequence homology to a MAG (Table 2). TEM imaging suggests that this phage is a podovirus (Figure 2). In a recent study of coliphages isolated from sewage and agricultural fecal samples, podoviruses were found to have the narrowest host-range [75]. Phages 527 and 1362 isolated here, however, exhibited much narrower ranges.

While myoviruses were the most common phage morphology observed, no consistent relationship between morphology and host lysis was observed. Most of the urinary strains tested for host range assays were lysogens themselves. The majority of bacteria in the urinary tract are lysogens [14], and recent research has found that lysogeny is prevalent in high-density bacterial populations, such as is the case with UTIs [33]. Furthermore, it is likely that superinfection immunity plays a significant role in determining coliphage-*E. coli* interactions. This too can have implications for phage therapy used to treat *E. coli*-associated UTIs [76], thus warranting continued investigation of temperate phages in the urinary tract.

Half of the temperate phages identified here did not exhibit sequence similarity to characterized phages, instead resulting in BLAST hits to MAGs (Table 2). This suggests that there remains a wealth of diverse coliphages in the human body that have yet to be characterized, although metagenomics has provided a glimpse into this diversity. Using ICTV tools, we identified the species exemplar sequence most similar to each of the phage genomes and assessed the intergenomic similarity. While we did not find instances in which the induced phage was a representative of one of these characterized species, we did find instances of phages induced from different *E. coli* strains, isolated from different individuals, that were representatives of the same species. In fact, we have characterized here two new *Peduovirus* species. Our comparison of the *Peduovirus* genomes finds protein sequence similarity despite non-collinearization of the phage genomes. This echoes a previous observation in which *Peduovirus* genomes isolated from clinical samples had mosaic architectures [77].

We would be remiss to not mention that the phages examined here are but a subset of predicted prophages in the 66 urinary *E. coli* strains (Supplemental Table S1 and Supplemental File S1). We selected for temperate phages capable of both infecting and lysing *E. coli* C and undergoing induction under the growth conditions used here. Testing cell-free supernatant on additional *E. coli* strains would likely identify additional temperate phages. For instance, Pacifico et al. used *E. coli* DSM 12242 to isolate phages from urine samples [77]. Induced coliphages from urinary *E. coli* isolates were purified using *E*. *coli* B, *E*. *coli* C, and *E*. *coli* K-12 by Crum et al. [15], and Almosuli et al. used *E. coli* B and UPEC *E. coli* CFT073 [33]. Furthermore, treatments such as UV light or mitomycin C would likely induce additional temperate phages. As we have previously shown, temperate coliphages can be induced from urinary *E. coli* strains through changes in a culture’s pH [57]. Even with the limited conditions employed here, a diverse collection of prophages infectious of urinary *E. coli* was catalogued, prompting subsequent studies into coliphages and their role in the urinary microbiota.

## Figures and Tables

**Table 1:** Bacterial host information for the 20 induced phages. Symptoms: No LUTS = no lower urinary tract symptoms, OAB = overactive bladder symptoms, UTI = Urinary tract infection, UUI = urge urinary incontinence.

Phage ID	Bacterial Strain	Bacterial Phylotype	Participant Symptom	No. Predicted Prophages
103	UMB0103	F	OAB	3
1091	UMB1091	B2_3_	UTI	5
149	UMB0149	A	OAB	5
527	UMB527	A	OAB	6
906	UMB906	B2_3_	UTI	5
923	UMB923	B1	UTI	4
931	UMB931	D	UTI	6
933	UMB0933	B2_2_	No LUTS	3
1160	UMB1160	B2_3_	UTI	6
1162	UMB1162	B2_3_	UTI	6
1180	UMB1180	B1	UTI	2
1225	UMB1225	D	UTI	2
1335	UMB1335	D	UTI	6
1362	UMB1362	B1	UTI	3
1526	UMB1526	B2_3_	UTI	2
3641	UMB3641	D	UUI	4
5814	UMB5814	B2_3_	UUI	2
6454	UMB6454	B2	UTI	5
6713	UMB6713	B2_3_	No LUTS	4
6890	UMB6890	B2_3_	UUI	4

**Table 2:** Sequence similarity to GenBank viral sequences and ICTV species exemplar virus sequences. This table summarizes each phage isolated, including the bacterial strain from which it was isolated and the phage record that has the closest sequence similarity to via BLAST. The genus and tail morphology of this nearest neighbor phage hit were determined either via the taxonomy listed in the BLAST record or from the literature (as noted in the table). ^a^ Per ICTV, this phage genome sequence is only available via the complete genome sequence of the *S. enterica* subsp. enterica serovar Typhimurium str. LT2 chromosome sequence, but it is located at position 2,844,298–2,877,981. ^b^ Values in bold exceed the genus threshold of 70%. ^c^ If no tail morphology is listed for the GenBank record, it is not included in the table. Tail morphology codes are as follows: M = myovirus, P = podovirus.

Phage ID	NCBI BLAST Results			ICTV TaxaBLAST Results		Predicted Tail Morphology ^c^
	Nearest Neighbor Strain (Genus; Accession No.)	Query Coverage	Percent Identity	Species Exemplar Virus (Genus; Accession No.)	Intergenomic Similarity ^b^	
103	MAG: Caudoviricetes sp. isolate MSP1011 (Uncharacterized; OR222389)	64%	80.37%	*Escherichia* phage 2H10 (*Glaedevirus*; LR595862.1)	30.0	
1091	Peduovirus P22H4 (*Peduovirus*; LR595869.1)	80%	97.66%	Enterobacteria phage fiAA91-ss (*Peduovirus*; KF322032.1)	**72.6**	M
149	*Escherichia* Phage 12W (*Peduovirus*; OM475434)	77%	96.80%	*Escherichia* phage P2_4E6b (*Peduovirus*; LR595885.1)	**80.1**	M
527	*Escherichia* phage phiv205-1 (*Lederbergvirus*; MN340231)	51%	96.32%	Enterobacteria phage HK620 (*Lederbergvirus*; AF335538.1)	35.8	P
906	Enterobacteria phage CUS-3 (*Lederbergvirus*; CP000711)	56%	96.05%	Enterobacteria phage HK620 (*Lederbergvirus*; AF335538.1)	35.9	P
923	*Escherichia* phage vB_EcoM-683R1 (Uncharacterized; ON470593)	72%	97.10%	*Salmonella* phage SEN34 (*Brunovirus*; KT630649.1)	31.3	M [55]
931	MAG: Bacteriophage sp. isolate 2344_22993 (Uncharacterized; OP074524)	92%	99.97%	*Escherichia* phage P2 (*Peduovirus*; KC618326.1)	4.7	
933	MAG: Caudoviricetes sp. isolate ctZgU1 (Uncharacterized; BK021527)	98%	95.66%	*Salmonella* phage PsP3 (*Eganvirus*; AY135486.1)	63.8	
1160	MAG: Bacteriophage sp. isolate 1228_163631 (Uncharacterized; OP073762)	100%	100%	*Escherichia* phage P2 (*Peduovirus*; KC618326.1)	0.3	
1162	Escherichia virus P2_2H4 (*Peduovirus*; LR595869)	82%	97.4%	*Escherichia* phage P2_2H1 (*Peduovirus*; LR595870.1)	**77.6**	M
1180	MAG: Bacteriophage sp. Isolate 0195_16858 (Uncharacterized; OP073076)	84%	97.8%	*Escherichia* phage P2_4E6b (*Peduovirus*; LR595885.1)	**80.8**	
1225	MAG: Bacteriophage sp. isolate 0195_16858 (Uncharacterized; OP073076)	83%	97.80%	*Escherichia* phage P2_4E6b (*Peduovirus*; LR595885.1)	**80.2**	
1335	MAG: Bacteriophage sp. isolate 1105_17178 (Uncharacterized; OP073677)	100%	99.81%	-	-	
1362	*Escherichia* phage vB_EcoP_ZX5 (*Uetakevirus*; MW722083)	100%	100%	*Escherichia* phage phiV10 (*Uetakevirus*; DQ126339.2)	59.5	P [56]
1526	*Escherichia* phage vB_EcoM-12474III (*Peduovirus*; MK907237)	74%	97.59%	*Yersinia* phage GMG73 (*Peduovirus*; OP880301.3)	45.2	M
3641	MAG: Bacteriophage sp. isolate 2344_22993 (Uncharacterized; OP074524)	81%	99.97%	*Salmonella* phage Fels2 (*Felsduovirus*; AE006468.2 ^a^)	1.2	
5814	MAG: Caudoviricetes sp. isolate ctc4i17 (Uncharacterized; BK019137)	70%	98.26%	*Salmonella* phage Fels2 (*Felsduovirus*; AE006468.2 ^a^)	0.1	
6454	MAG: Bacteriophage sp. isolate 0195_16858 (Uncharacterized; OP073076)	83%	97.81%	*Escherichia* phage P2_4E6b (*Peduovirus*; LR595885.1)	**80.1**	
6713	MAG: Bacteriophage sp. isolate 1105_17178 (Uncharacterized; OP073677)	79%	100%	-	-	
6890	*Escherichia* phage D108 (*Muvirus*; GQ357916)	96%	96.52%	*Escherichia* phage Mu (*Muvirus*; AF083977.1)	68.3	M

## Data Availability

Genome accession numbers for the *E. coli* genomes are included in Supplemental Table S1. Genome accession numbers for the induced phages are included in Supplemental Table S2.

## Supplementary Materials

The following supporting information can be downloaded at: https://www.mdpi.com/article/10.3390/v18020179/s1, Table S1. Details about 66 urinary *E. coli* isolates and their genomes; Table S2. Details about the 20 induced *E. coli* phage genomes; Table S3. Acrs predicted for the induced *E. coli* phage genomes; Figure S1. Example of dilutions of induced prophages on *E. coli* lawns in which the plaques are not clear, but rather they appear cloudy; Figure S2. VIRIDIC heatmap comparing the phage genomes to TaxaBLAST-identified ICTV species exemplar virus sequences. The upper triangle lists the intergenomic similarity (%) while the lower triangle lists the fraction (between 0 and 1) of the genome that aligned. Three measures are provided for the genome alignment: the aligned fraction genome 1 (row genome), genome length ratio (for the two genomes in the pair), and aligned fraction genome 2 (column genome); File S1. PHASTER predicted prophage sequences for *E. coli* genomes from which phages were isolated.
