# Supplementary material for: Diverse Temperate Coliphages of the Urinary Tract
Source: Viruses. 2026 Jan 29;18(2):179. doi: 10.3390/v18020179 (PMC12945033; doi:10.3390/v18020179)
Supplement: Supplementary file 1 [file viruses-18-00179-s001.zip › Supplemental_Table S1.pdf]

Supplemental Table 1. Details about 66 urinary *E. coli* isolates. Serotype, phylotype, and participant symptoms obtained from Garretto *et al*. [3]

Strain	Serotype	Phylotype	Participant Symptom	RefSeq Assembly Accession #	No. Prophages	No. CRISPR Spaces
103	O1:H6	F	OAB	GCF_003892645	3	2
149	O86:H30	A	OAB	GCF_003892555	5	2
276	O6:H1	$B2_3$	OAB	GCF_003892545	1	0
527	O11:H4	A	OAB	GCF_003892535	6	1
731	O6:H31	$B2_3$	OAB	GCF_003892485	3	0
906	O25:H4	$B2_3$	UTI	GCF_003886695	5	0
923	O8:H49	B1	UTI	GCF_003892635	4	2
928	O75:H5	$B2_3$	no LUTS	GCF_003892445	3	0
931	O17/44:H18	D	UTI	GCF_003886495	6	2
933	O21:H5	$B2_2$	no LUTS	GCF_003886675	3	0
934	O11:H16	D	UTI	GCF_003892475	3	2
939	O6:H1	$B2_3$	no LUTS	GCF_003885295	1	0
949	O17/44:H45	D	UTI	GCF_003892435	5	2
1012	O16:H5	$B2_3$	UTI	GCF_003886455	2	0
1091	O16:H5	$B2_3$	UTI	GCF_003886445	5	0
1093	O17/77:H18	D	UTI	GCF_003885215	4	2
1160	O75:H5	$B2_3$	UTI	GCF_003892605	6	0
1161	O25:H4	$B2_3$	UTI	GCF_003885195	2	0
1162	O2:H6	$B2_3$	UTI	GCF_003892455	6	2
1180	O21:H21	B1	UTI	GCF_003892375	2	2
1193	O4:H5	$B2_2$	UTI	GCF_003892595	2	0
1195	O13:H4	$B2_3$	UTI	GCF_003886435	7	0
1202	O8:H10	$B2_3$	UTI	GCF_003886395	1	0
1220	O6:H31	$B2_3$	UTI	GCF_003886385	4	0
1221	O2:H6	B2	UTI	GCF_003885055	6	2
1223	O17/44:H18	D	UTI	GCF_003886375	2	2
1225	H34; No O	D	UTI	GCF_003886735	2	1
1228	O25:H4	B2	UTI	GCF_003886655	4	0
1229	O4:H5	$B2_2$	UTI	GCF_003886345	1	0
1284	O25:H4	$B2_3$	UTI	GCF_003892355	4	0
1285	O13:H4	$B2_3$	UTI	GCF_003886635	2	0
1335	O2:H18	D	UTI	GCF_003886615	6	2
1337	O50/2:H18	D	UTI	GCF_003886325	6	2
1346	O166:H15	D	UTI	GCF_003886295	2	1
1347	O166:H15	D	UTI	GCF_003886285	2	1

Strain	Serotype	Phylotype	Participant Symptom	RefSeq Assembly Accession #	No. Prophages	No. CRISPR Spaces
1348	O75:H5	$B2_3$	UTI	GCF_003886275	5	0
1354	O166:H15	D	UTI	GCF_003886225	2	1
1356	H15; No O	D	UTI	GCF_003886245	2	1
1358	O147:H21	A	UTI	GCF_003886195	1	2
1359	H15; No O	D	UTI	GCF_003886185	2	1
1360	O6:H1	$B2_3$	UTI	GCF_003886565	1	0
1362	H7; No O	B1	UTI	GCF_003886175	3	1
1526	O6:H31	$B2_3$	UTI	GCF_003886105	2	0
1727	O17/44: H18	D	UUI	GCF_003886135	3	2
2019	O19:H4	A	UUI	GCF_003886115	3	3
2055	O59:H23	B1	UUI	GCF_003886095	2	2
2328	O17/77:H31	A	UUI	GCF_003886545	1	2
3538	O25:H4	$B2_3$	UUI	GCF_003886535	3	0
3641	O17/44:H18	D	UUI	GCF_003885305	4	2
3643	O6:H31	$B2_3$	UUI	GCF_003885095	6	0
4656	O1:H7	$B2_3$	UTI	GCF_003886515	5	2
4716	O22:H1	$B2_3$	UUI	GCF_003885995	6	0
4746	H1; No O	$B2_3$	UUI	GCF_003886045	4	0
5337	H15; No O	D	UUI	GCF_003886035	2	1
5814	O6:H31	$B2_3$	UUI	GCF_003886015	2	0
5924	O75:H5	$B2_3$	UTI	GCF_003886005	3	0
5978	O75:H5	$B2_3$	UTI	GCF_003885915	4	0
6454	O2:H7	$B2_3$	no LUTS	GCF_003885245	5	2
6471	O7:H15	D	UTI	GCF_003885155	2	1
6611	O18:H7	$B2_3$	no LUTS	GCF_003885875	3	2
6653	O11:H18	D	UTI	GCF_003885965	3	2
6655	O75:H31	$B2_3$	UUI	GCF_003885255	4	0
6713	O2:H7	$B2_3$	no LUTS	GCF_003885145	4	2
6721	O11:H18	D	UTI	GCF_003885125	3	2
6890	H31; No O	$B2_3$	UUI	GCF_003885035	5	0
7431	O11:H18	D	UTI	GCF_003885225	4	2
