# Supplementary material for: Diverse Temperate Coliphages of the Urinary Tract
Source: Viruses. 2026 Jan 29;18(2):179. doi: 10.3390/v18020179 (PMC12945033; doi:10.3390/v18020179)
Supplement: Supplementary file 1 [file viruses-18-00179-s001.zip › Supplemental_Table S2.pdf]

**Supplemental Table 2.** Details about the 20 induced *E. coli* phage genomes.

| Phage ID | Genome Accession No. | Genome length (bp) | No. Coding Regions |
|---|---|---|---|
| 103 | *Awaiting from NCBI* | 36094 | 61 |
| 1091 | *Awaiting from NCBI* | 32086 | 57 |
| 149 | *Awaiting from NCBI* | 34401 | 53 |
| 527 | *Awaiting from NCBI* | 41568 | 83 |
| 906 | *Awaiting from NCBI* | 36133 | 81 |
| 923 | *Awaiting from NCBI* | 15102 | 25 |
| 931 | *Awaiting from NCBI* | 34530 | 60 |
| 933 | *Awaiting from NCBI* | 19775 | 31 |
| 1160 | *Awaiting from NCBI* | 10610 | 21 |
| 1162 | *Awaiting from NCBI* | 31890 | 51 |
| 1180 | *Awaiting from NCBI* | 33724 | 50 |
| 1225 | *Awaiting from NCBI* | 34421 | 55 |
| 1335 | - | - | - |
| 1362 | *Awaiting from NCBI* | 37628 | 61 |
| 1526 | *Awaiting from NCBI* | 9876 | 18 |
| 3641 | *Awaiting from NCBI* | 39312 | 68 |
| 5814 | *Awaiting from NCBI* | 26452 | 30 |
| 6454 | *Awaiting from NCBI* | 34346 | 54 |
| 6713 | - | - | - |
| 6890 | *Awaiting from NCBI* | 22703 | 42 |

- genomes to fragmented for submission.
