# Supplementary figures and images for: Diverse Temperate Coliphages of the Urinary Tract
Source: Viruses. 2026 Jan 29;18(2):179. doi: 10.3390/v18020179 (PMC12945033; doi:10.3390/v18020179)

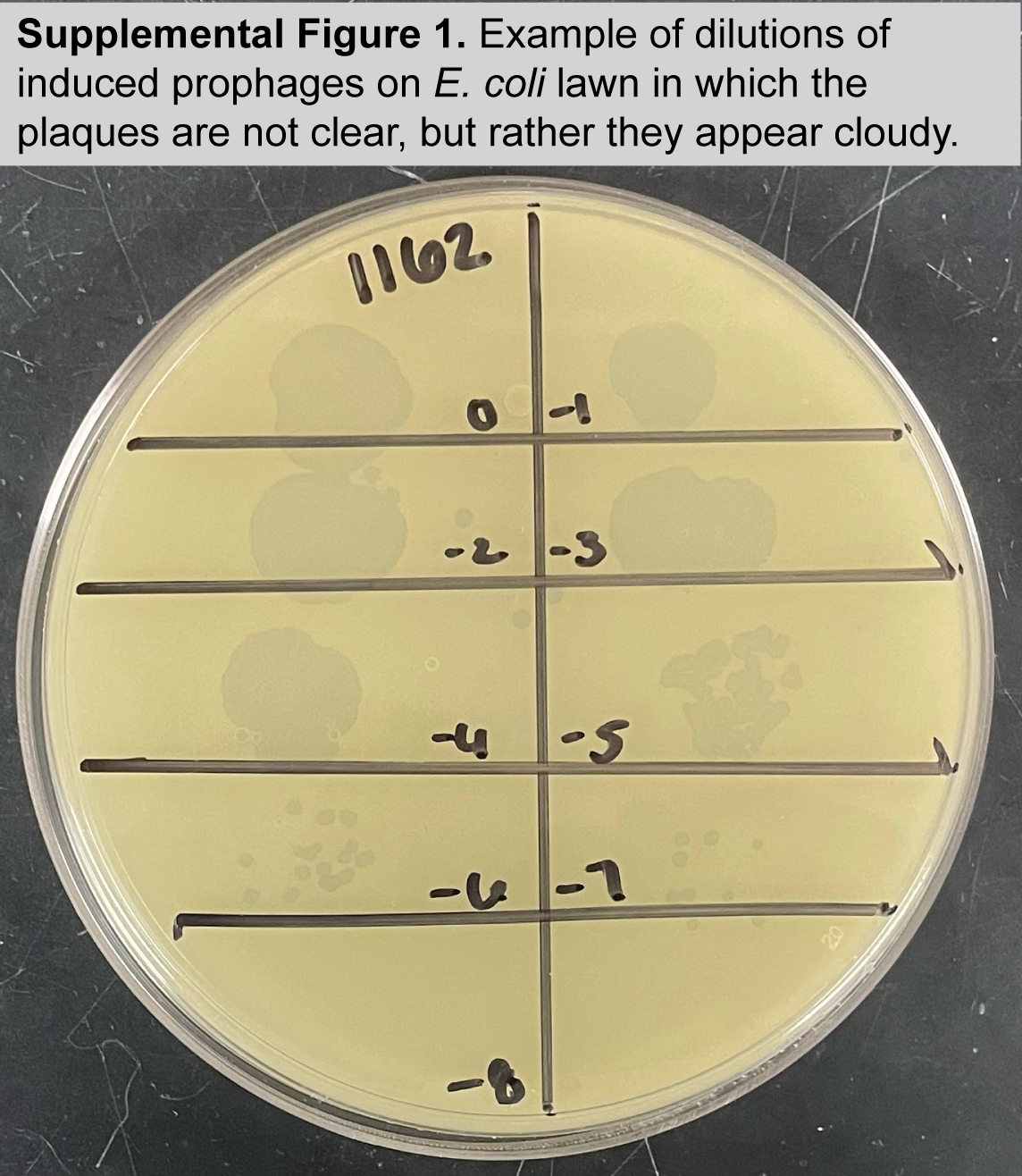

Supplement: Supplementary file 1 [file viruses-18-00179-s001.zip › Supplemental_Figure S1.tif]

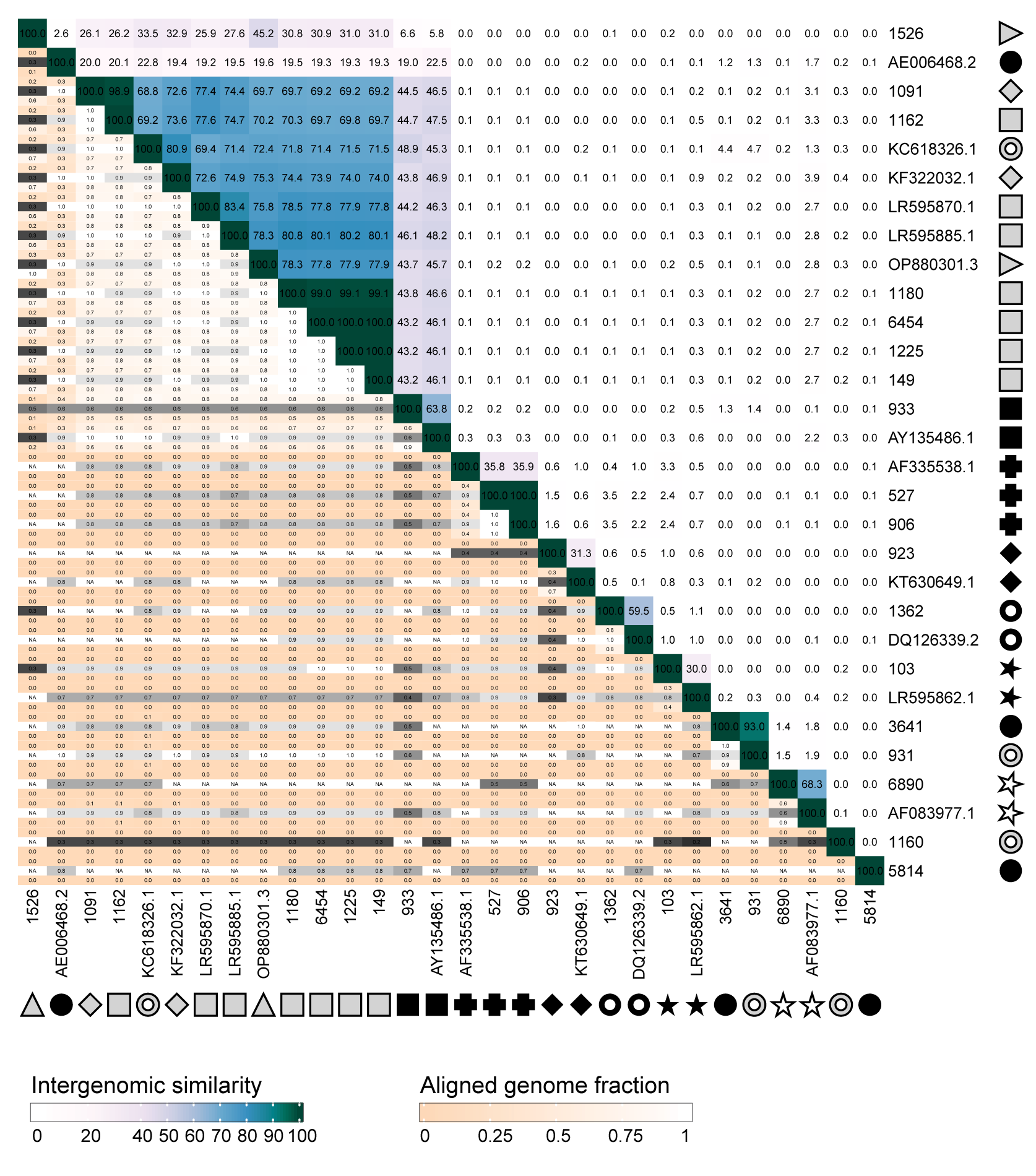

Supplement: Supplementary file 1 [file viruses-18-00179-s001.zip › Supplemental_Figure S2.tif]
